# Investigating the Relationship Between the Emulsification Parameters and Physical–Chemical Properties of Poly(D,L-lactic acid) Particles for Dermal Fillers

**DOI:** 10.3390/polym16233395

**Published:** 2024-12-01

**Authors:** Chen-Ying Su, You-Cheng Chang, Bo-Rong Lu, Hsu-Wei Fang

**Affiliations:** 1 Department of Chemical Engineering and Biotechnology, National Taipei University of Technology, 1, Sec. 3, Zhongxiao E. Rd., Taipei 10608, Taiwan; chenying.su@mail.ntut.edu.tw (C.-Y.S.); youchengchang@mail.ntut.edu.tw (Y.-C.C.); t112738002@ntut.org.tw (B.-R.L.); 2 High-Value Biomaterials Research and Commercialization Center, National Taipei University of Technology, 1, Sec. 3, Zhongxiao E. Rd., Taipei 10608, Taiwan; 3 Institute of Biomedical Engineering and Nanomedicine, National Health Research Institutes, No. 35, Keyan Road, Zhunan Town, Miaoli 35053, Taiwan

**Keywords:** poly(D,L-lactic acid) acid particle, dermal filler, emulsification, carboxymethyl cellulose, soft-tissue augmentation, reconstitution

## Abstract

Poly(L-lactic acid) (PLLA) and poly(D,L-lactic acid) (PDLLA) particles have been applied as dermal fillers for soft-tissue augmentation because they can induce foreign-body reactions, resulting in fibroblast proliferation and collagen formation. Although PLLA and PDLLA fillers are safe and biocompatible, clinical complications such as nodules and granulomas have been reported, possibly due to incomplete reconstitution. PDLLA particles were prepared via emulsification in this study, and three stirring speeds were investigated when adding PDLLA into carboxymethyl cellulose solution. The particle size, molecular weight of PDLLA, optical rotation, pH value, osmotic pressure, and reconstitution time were analyzed. A rabbit dorsal ear model was established to evaluate the soft-tissue augmentation of a commercial PDLLA filler. The results demonstrated that the stirring speed affected the particle size, but not other physical–chemical properties of the PDLLA particles. All the PDLLA particles were reconstituted in less than 7 min, which is faster than the process for the other commercial PDLLA dermal filler products. In addition, the PDLLA particles could induce inflammation and fibroblast proliferation. Although the PDLLA particles generated in this study have not yet been investigated in vivo, the results demonstrated here suggest their potential for application as dermal fillers.

## 1. Introduction

Aging is a process that affects the body’s organs and functions. The majority of people notice the aging of the face, including the loss of subcutaneous fat, skin laxity, and wrinkles [1]. In order to solve facial aging, dermal fillers have been applied extensively [2]. Dermal fillers can be divided into permanent, semi-permanent, and temporary types, depending on their biodegradability. The materials used for permanent dermal fillers include polymethylmethacrylate (PMMA), polyacrylamide, or silicone, which are safe and effective clinically [3,4,5]. The current semi-permanent dermal filler materials include hydroxylapatite microspheres in sodium carboxymethyl cellulose, polylactic acid (PLA), autologous fat, and even adipose-derived stem cells [1,6,7]. Some temporary dermal fillers are hyaluronic acid and calcium hydroxyapatite [8,9]. Permanent and semi-permanent dermal fillers can induce inflammatory and minor foreign-body reactions, resulting in fibroblast proliferation and collagen synthesis [10,11]. Semi-permanent dermal fillers can be absorbed for up to 24 months, depending on the amount and type of material injected, while permanent fillers can last for more than 5 years [1]. Therefore, they are suitable for deeper wrinkle and fold treatments. Temporary dermal fillers usually cause immediate augmentation after injection. The duration of degradation, which may range from 6 to 18 months, is dependent on the injection location [12].

Among all the dermal fillers, semi-permanent ones might be the most popular choice; they can maintain efficacy for longer than temporary fillers and can biodegrade faster than permanent fillers, which may reduce some clinical risks. The currently used semi-permanent dermal fillers include Radiesse, Sculptra, AestheFill, Repart PLA, Gana V, and NeoFilera. Radiesse is composed of calcium hydroxylapatite microspheres suspended in a gel containing sodium carboxymethyl cellulose and glycerol [1]. The rest of the semi-permanent filler products are mainly composed of PLA and sodium carboxymethylcellulose (CMC), with or without mannitol. The PLA particles in AestheFill, Repart PLA, and NeoFilera are made of poly(D,L-lactic acid) (PDLLA), while Sculptra and Gana V are composed of poly(L-lactic acid) (PLLA) [2]. Although PDLLA and PLLA exhibit some differences, such as their glass transition and melting temperatures, tensile strength, and degradation time, both can stimulate the fibroblasts to synthesize collagen [13,14,15]. The mechanism of how PLA particles stimulate collagen formation is that the immune cells consider PLA as a foreign body and inflammation occurs, resulting in PLA-induced collagen synthesis [10,16]. In order to influence the biological responses, it has been shown that both the size and surface morphology of PLA particles are critical factors. When the size of particles is smaller than 20 μm, phagocytosis might occur by macrophages, resulting in giant cell formation and granulomatous inflammation [17]. However, particle size should be smaller than 100 μm to easily pass through the needle [2]. Research has demonstrated that the rough surfaces and irregular shapes of particles cause the clustering of allogeneic giant cells, suggesting that those with smooth surfaces and regular shapes are more suitable for dermal fillers [18]. The average PLA particle size of AestheFill is 27 ± 17 μm, while it is 28 ± 16 μm for Repart, 42 ± 22 μm for Gana V, and 52 ± 29 μm for Sculptra—all are within the range of 20 and 100 μm and display smooth surfaces [2], indicating they are suitable for dermal fillers.

Although PLA has been proven to be safe and biocompatible by the United States Food and Drug Administration, some clinical complications have been reported. The most common short-term adverse reactions of injecting PLA dermal fillers are localized ecchymosis and edema, while long-term complications include nodules, subcutaneous papules, and granulomas [7,19,20]. Some case reports have demonstrated that nodules and subcutaneous papules might be caused by excessive deposits or the inadequate dispersion of PLA fillers, as well as an insufficient volume of dilution [19,21]. Therefore, improving the dispersion of PLA particles or increasing the dilution volume might be able to eliminate the clinical complications caused by PLA dermal filler injection. Indeed, fewer adverse events occurred once more diluents were mixed with PLA particles to make a thinner suspension [22]. The current instruction for injecting PLLA fillers is adding 5 mL sterile water into a vial of PLLA powder and letting the vial sit for at least 2 h until complete hydration [23]. Vigorous shaking is not recommended during the hydration process, but gently agitating the vial immediately before injection is needed. Adding 1.4 or 8 mL of sterile water into a vial of PDLLA powder is instructed to inject the PDLLA product, and PDLLA can be reconstituted either using a shake generator or handshaking. However, it usually takes more than 30 min until the PDLLA powder is completely homogenous [24]. Therefore, it will save doctors time and reduce complications if the PLA particles are dispersed evenly and quickly during reconstitution. The potential solution for shortening reconstitution is still unclear, but it has been suggested that the critical step is to prevent CMC aggregation during reconstitution, resulting in evenly dispersed PLA particles [24]. Consequently, understanding how PLA and CMC incorporate with each other during the preparation process tends to be the key solution.

PLA dermal fillers need to be stored as a powder to prevent the hydrolytic degradation of PLA and CMC [25,26]. During the manufacturing of the currently used PDLLA fillers, microspheres are prepared first and then suspended in a CMC solution to be lyophilized into a powder [24,27]. As PLA is hydrophobic and CMC is hydrophilic, emulsification, precipitation, direct compositing, or microfluidic techniques should be applied to produce PLA particles [28]. Emulsification is dissolving PLA in an organic solvent and emulsifying in an aqueous solution containing an emulsifier. Emulsification is a simple and fast method that controls various particle sizes by adjusting the stirring rate, the viscosity or phase volumes of organic/aqueous phases, the concentration of the PLA/CMC/emulsifier, and the presence of surfactant/stabilizer [28]. The determination of emulsification parameters makes it easier to scale up by manufacturing multiple batches in parallel lines. Hence, we chose emulsification to generate PDLLA particles in this study. As the particle size of the current PLA dermal filler products ranges from 30 to 70 μm [2], the effects of various stirring speeds on the size of PDLLA particles were investigated. The physical–chemical properties of PDLLA particles, such as the molecular weight of PDLLA, particle morphology and size, optical rotation, pH value, osmotic pressure, and reconstitution, were examined. A commercial PDLLA dermal filler, NeoFilera, was used as a control. NeoFilera was injected into the dorsal ears of rabbits in order to evaluate whether this animal model can be used to test PDLLA particle applications in the future.

## 2. Materials and Methods

### 2.1. The Manufacturing Process of Polylactic Acid Particles

The preparation of poly(D,L-lactic acid) (PDLLA) particles consisted of two phases: oil and water phases. For the oil phase, 10% (*v*/*v*) PDLLA solution was prepared by adding D,L-form PLA powder (Green Square Materials Inc., Hsinchu, Taiwan; molecular weight 80,000–90,000 Daltons) into acetone (Sigma-Aldrich, St. Louis, MO, USA). For the water phase, 1 g of carboxymethyl cellulose (CMC, Sigma-Aldrich; molecular weight 700,000–800,000 Daltons) was dissolved in 400 milliliters (mL) of ultrapure water. In a 500 mL beaker, 45 mL of PDLLA solution was added to 350 mL of CMC solution and stirred for 5 hours (h) with a magnetic stir bar (0.5 cm in diameter and 5 cm in length) at room temperature. Stirring speeds of 100, 200, or 400 revolutions per minute (rpm) were used in this study. The mixed solution was kept at −80 °C for 12 h, then freeze-dried for 48 h. The dried powders were dissolved in a 4% mannitol solution (Sigma-Aldrich) and then lyophilized to obtain PDLLA particles for further analysis. The currently used PDLLA dermal filler product (NeoFilera, Diamond Biotechnology Co. Ltd., Taipei, Taiwan; Thailand Medical Device certificate 67-2-1-2-0005765) was used as a control in this study.

### 2.2. Morphological Observation of PDLLA Particles

The surface morphology of various PDLLA particles was imaged using a field emission scanning electron microscope (JSM-7610F, JEOL, Peabody, MA, USA) at 15 kV with a Schottky field emission electron gun at a particular distance. A total of 200 milligrams (mg) of the PDLLA particles generated using various stirring speeds or NeoFilera were dissolved in 8 mL of sterile distilled water. The suspended solution was then kept at −80 °C for 24 h and freeze-dried for 24 h. The dried particles were fixed to a metal support and coated with gold with a sputter coater (Ion Sputter E101, Hitachi, Tokyo) for imaging. The micrograph images were taken at two different magnifications: 100× and 500×. The lengths of random PDLLA particles generated from each stirring speed were measured using Image J software (version 1.54), and 100 particles were measured for each group to obtain the average particle length.

### 2.3. Measurement of Molecular Weight

The molecular weights of PDLLA and CMC in the final PLA particles prepared with various stirring speeds, as well as NeoFilera, were measured using gel permeation chromatography (GPC, Malvern Viscotek 270 max). A total of 200 mg of the final PDLLA particles was dissolved in 8 mL of sterile distilled water. The PDLLA phase was separated from the CMC phase by centrifuging at 3000 rpm for 10 min. Next, 4 mg of the PDLLA phase (lower phase) was dissolved in 1 mL of tetrahydrofuran (Sigma-Aldrich), and 4 mg of the CMC phase was dissolved in 1 mL of dimethylformamide (Sigma-Aldrich). Each phase was sonicated for 1 h or until there was no precipitation and then filtered through a 0.22 μm membrane filter at room temperature. The filtered PDLLA or CMC solution was injected during GPC at 1 mL/min when the temperature was 65 °C. Three independent PDLLA particles from each stirring speed were measured.

### 2.4. Characteristics of Physical–Chemical Properties

**Figure 1:**
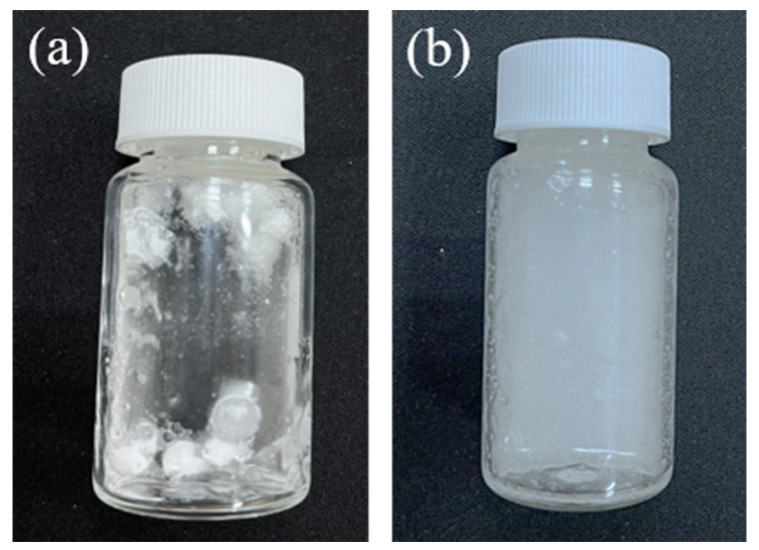
A picture of reconstitution testing (**a**) when distilled water was added to a vial of PDLLA particles immediately or (**b**) after all the particles had dissolved and dispersed.

For this study, four physical–chemical properties of the PDLLA particles generated at the various stirring speeds and NeoFilera were characterized, including optical rotation, the pH value, reconstitution, and osmotic pressure. A total of 200 mg of PDLLA particles or NeoFilera was dissolved in 5 mL of acetone to measure optical rotation using a Polarimeter (P-2000, Jasco, Easton, MD, USA). Similarly, a total of 200 mg of PDLLA particles or NeoFilera was dissolved in 8 mL of sterile distilled water to measure the pH value using a pH meter (Eutech pH meter 510, Eutech Instruments Pte Ltd, Singapore). For the osmotic pressure measurement, 200 mg of PDLLA particles or NeoFilera was dissolved into 8 mL of distilled water. After gently shaking and keeping the sample still for 10 min, osmotic pressure was measured using an osmometer (Fiske Associates 210, Advanced instruments, Norwood, MA, USA). For reconstitution, 1.4 mL or 8.0 mL of distilled water was added to 200 mg of PDLLA particles or NeoFilera (Figure 1a). The mixture was vortexed at 2700 rpm, and the reconstitution time was recorded until all the particles had dissolved and dispersed evenly (Figure 1b). A current commercial PDLLA dermal filler (AestheFill) is designed to be suspended in 1.4 or 8 mL of sterile water for deep-to-shallow wrinkle correction [29]; thus, these two volumes of water were selected for reconstitution analysis. Three independent samples from each stirring speed test were measured.

### 2.5. Fourier-Transform Infrared Spectroscopy Analysis

The functional groups of the PDLLA particles generated from various stirring speeds or NeoFilera were identified using a Fourier-transform infrared spectroscope (FTIR, Spotlight 200i Sp2 with AutoATR System, Perkin Elmer, Shelton, CT, USA). A total of 100 mg of PDLLA or NeoFilera particles was ground in a mortar and pressed into tablets using a tableting machine for FTIR measurements. The FTIR spectra were recorded over a wavenumber range from 400 to 4000 cm^−1^, and 32 scans were run.

### 2.6. Performance Test of PLA Particles with Rabbit Dorsal Ear Model

To investigate the effects of different stirring speeds on collagen formation and fibroblast proliferation in the PDLLA particles, an in vivo performance test was established using the currently used PDLLA dermal filler product (NeoFilera). Male New Zealand rabbits weighing more than 2 kilograms (kg) were used in this study. The experimental procedure was performed according to the Institutional Animal Care and Use Committee (IACUC) of Master Laboratory Co., Ltd. (Hsinchu, Taiwan) (IACUC approval no. 22T10-06). Prior to the experiment, the dorsal fur of both the rabbits’ ears was clipped with an electric animal shaver. Zoletil (10 mg/kg) and xylazine (10 mg/kg) were administered as general anesthesia into the muscles of the rabbits. A total of 200 mg of NeoFilera was dissolved in 8 mL of sterile distilled water, and 0.1 mL of NeoFilera solution r normal saline was subcutaneously injected into the dorsal ears of each rabbit using a 26-gauge needle. Both NeoFilera solution and normal saline were injected into the left or right ears of each rabbit. Six rabbits were sacrificed after 12 weeks post-injection, and another six animals were sacrificed after 26 weeks.

### 2.7. Histological Analysis

The specimens were harvested and preserved in 10% neutral buffered formalin 24 h after sacrifice, followed by decalcification for 36 h The samples were subjected to trimming, fixation, dehydration, embedding, and slicing to obtain 3–4 slides. The slices were stained with hematoxylin and eosin (H&E) or Masson’s trichrome (MT) to examine collagen formation and fibroblast proliferation under a microscope.

Histopathological scoring was used to grade the various parameters that reflect the degree or extent of injury, inflammation, and the hosts’ response to the injected samples. Scores between 0.0 and 2.9, 3.0 and 8.9, and 9.0 and 15.0, and those over 15.0 were considered minimal or reflected the following reactions: none, slight, moderate, and severe, respectively.

### 2.8. Statistical Methods

For the animal experiment, all values are expressed in the form of mean ± standard deviation. The unpaired Student’s *t*-test (SPSS, version 22.0) was used to compare the animals injected with normal saline or NeoFilera particles. *p* < 0.05 is considered to be significant.

## 3. Results and Discussion

### 3.1. Influence of Stirring Speeds

**Figure 2:**
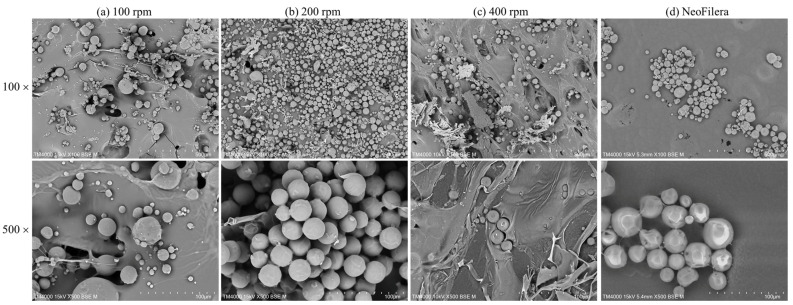
Scanning electronic microscope images of PDLLA particles that were generated at stirring speeds of 100 rpm (**a**), 200 rpm (**b**), or 400 rpm (**c**), as well as a commercial NeoFilera PDLLA product (**d**). Scale bar shows 50 μm with 100× magnification and 10 μm with 500× magnification.

Three different stirring speeds were tested when mixing oil-phase PDLLA and water-phase CMC. Particle morphology was observed after freeze-drying. When the stirring speed was 100 rpm, the averaged particle size was 57.29 ± 16.89 µm (Figure 2a). Some large sheets were observed, suggesting that the CMC solution was completely mixed with the PDLLA droplets during emulsification. A similar phenomenon was also observed when the stirring speed was 400 rpm; some large sheets were mixed with the PDLLA particles. The average size was 15.53 ± 6.39 µm, and the majority of the PDLLA–CMC mixture did not form particles, resulting in a high loss rate as well as a potential induction of phagocytosis (Figure 2c). In contrast, the PDLLA particle size was 29.64 ± 8.44 µm, and there were no PDLLA–CMC sheets observed at 200 rpm (Figure 2b), indicating that the most optimized stirring speed for the mixed PLA and CMC phases was 200 rpm. The morphology of NeoFilera PDLLA particles demonstrated that the particles were round and distributed evenly in size (Figure 2b). The average size was 24.51 ± 0.86 μm, which is close to that of the PDLLA particles generated at 200 rpm (Figure 2b).

### 3.2. The Physical–Chemical Properties of Various PDLLA Particles

To confirm whether the PDLLA polymers were disrupted or hydrolyzed during manufacturing, the molecular weight of PDLLA in the final particles was analyzed by GPC. The original molecular weight of PDLLA was 80,000–90,000 Daltons, and the range of the PDLLA averaged molecular weight was between 84,386 and 84,525 Daltons, regardless of the stirring speed (Table 1). The commercial NeoFilera PDLLA particles also showed a similar result, and the original GPC curves of each sample are shown in Supplementary Figure S1. The optical rotation result showed that the PDLLA particles and NeoFilera were larger than +0.21, indicating all the samples maintained the D,L-form of PLA (Table 1). These results suggested that the manufacturing process used here did not change the basic properties of PDLLA.

**Figure 3:**
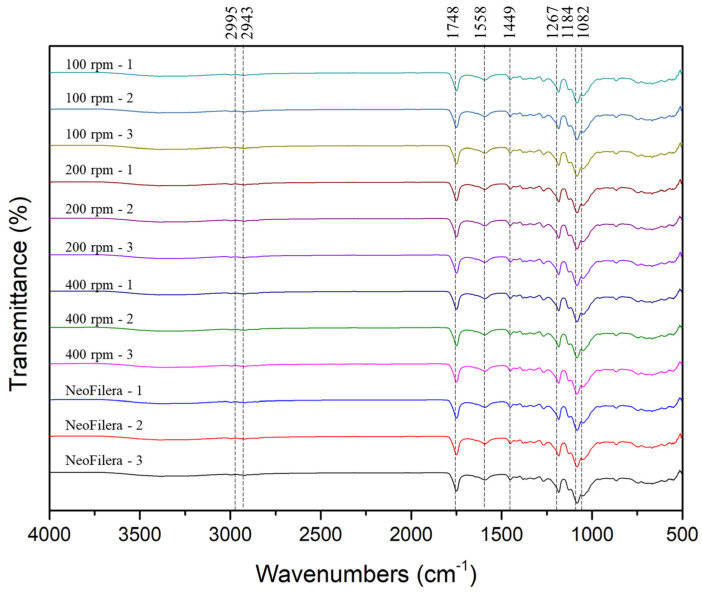
Characteristics of functional groups in various PDLLA particles (100, 200, or 400 rpm) and commercial NeoFilera PDLLA particles.

The results demonstrate that, regardless of the stirring speed, the pH value of all the PDLLA particles was neutral, and the osmotic pressure was around 280 mOsm/kg. In addition, all the PDLLA particles were reconstituted completely in 6.5 or 3 min when adding 1.4 mL or 8.0 mL of distilled water, respectively. In addition, functional groups of various PDLLA particles and NeoFilera were analyzed. The carbon–hydrogen (C-H) and ester (COOH) groups of PLA were observed at 2943–2995 and 1746 cm^−1^, respectively [30]. The functional groups of CMC include hydroxyl (OH), carbonyl (C=O), C-H alkanes, and C-O carboxylic acid/ester groups, present at around 3347, 2934, 1648–1710, and 1026–1227 cm^−1^ [31]. The FTIR results show that the function groups of C-H and COOH were observed at 2995, 2943, and 1748 cm^−1^, suggesting that these characteristic peaks were from PLA (Figure 3). The functional groups of OH, C=O, C-H alkanes, and C-O were present at 3200–3600, 1588, 1449, and 1082–1267 cm^−1^ (Figure 3), which is similar to the functional groups of CMC. Therefore, the main ingredients of PDLLA particles generated by various stirring speeds and NeoFilera PDLLA particles are PLA and CMC.

The results suggest that the physical–chemical properties of the PDLLA particles were similar regardless of the stirring speed. The physical–chemical properties of NeoFilera, a current commercial PDLLA dermal filler product, were also close to the results of PDLLA particles generated from three stirring speeds (Table 1), demonstrating the PDLLA particles produced at 100 and 200 rpm in this study might be potentially developed as dermal fillers.

Different PLA particle sizes can be generated by controlling the stirring rate, the oil and water phase volumes, the stirring rate, and other factors during emulsification [28]. In this study, a PDLLA solution was added to a CMC solution, and PDLLA droplets were dispersed as the two solutions were immiscible during emulsification. CMC is a good emulsifier due to its unique structure; some hydroxyl groups on the cellulose backbone are replaced with carboxymethyl groups, resulting in water solubility [32]. Hydroxyl groups that are not replaced on the backbone of CMC can interact with molecules in the solution via intra/intermolecular hydrogen bonding, resulting in aggregation [33]. It is possible that the PDLLA–CMC particles were formed by specific intramolecular hydrogen bonding during emulsification, and the particle sizes were affected by the stirring speeds. Faster stirring can generate a higher shear force, and many researchers have shown that this reduces the size of polymer droplets or the dispersed phase of polymer blends. Sui et al. have demonstrated that the size of PLA droplets dispersed in a polypropylene matrix reduced from 1.09 to 0.68 μm when the rotation speed was increased from 100 to 900 rpm [34]. Shimizu et al. also showed that 11 polyamide droplets with a diameter of 20–100 nm were evenly dispersed in a poly(vinylidene fluoride) phase when the screw rotation speed was 1200 rpm [35]. The size of PDLLA was reduced from 57 to 15 μm when the stirring speed was increased from 100 to 400 rpm in this study (Figure 2). The results suggest that the stirring speed is a critical parameter for determining the size of PDLLA particles. In addition, the particle sizes when the stirring speeds were 100 and 200 rpm were within 20 and 100 μm, which would not induce phagocytosis and could go through a needle easily [2,17]. However, the whole stirring process took 5 h at room temperature, and this may be too long for large-scale medical device manufacturing. A previous study has also shown that the temperature of the stirring process affects the size of PLA droplets [34]; therefore, the temperature should be increased.

When the PDLLA particles were lyophilized into a powder form, sterile water was added for reconstitution to analyze the physical–chemical properties caused by the different stirring speeds. The results showed that all the PDLLA particles displayed similar characteristics regardless of the stirring speed. The molecular weight of PLA in all the PDLLA particles remained within the range from 80,000 to 90,000 Daltons, which is the same as that for raw PDLLA. This result suggested that PDLLA did not decompose during emulsification. The optical rotation of PLLA and PDLA depends on the types of solution and concentration [35]. For example, Feng et al. have shown that the optical rotations of neat PLLA and PDLA are −266.6° and +266.0° in a dichloromethane solution [36]. The specific optical rotation values of pure PLLA and PDLA in chloroform are −156° and +156° [37]. Despite the solvent used for measuring the optical rotation of PLA, the value of PLLA is usually negative, while that for PDLA is positive. The optical rotation of PDLLA is determined by the percentage of PLLA and PDLA in the polymers; this value was close to +266.0° when the mass fraction of D-lactide was increased [36]. The results of optical rotations in this study were between +0.21 and +0.26, suggesting that both L-lactide and D-lactide were present. However, the exact percentages of L- and D-lactide in PDLLA used here require further investigation. The pH values of all the PDLLA particles were close to seven, indicating that they are suitable for clinical use. The osmotic pressure value was around 280 mOsm/kg, which is close to serum osmolality (275–295 mOsm/kg) [38]. This suggests that PDLLA particles generated by various stirring speeds should not cause hyperosmoticity or hypotonicity when injected into subcutaneous areas.

As mentioned in the Introduction, it takes more than 30 min for the commercial PDLLA dermal filler (AestheFill) to be reconstituted in sterile water using the vortex method [24]. In contrast, the PDLLA particles generated here were all reconstituted in 1.4 or 8 mL of sterile water in less than 7 or 3 min, respectively (Table 1). In order to avoid nodules developing due to the incomplete resuspension of PLA fillers [21], reconstitution should be encouraged to obtain a homogeneous suspension without any particle aggregates. The first step of reconstitution should be dissolving CMC particles into the solution; the second step should be separating all the PDLLA particles, followed by suspending the PDLLA particles evenly in a solution. The PDLLA particles of AestheFill are obtained using the solvent spray technique, which is suspended in a CMC solution and then lyophilized into the final product [27]. CMC may only be coated on the surface of the PDLLA particles in AestheFill without any intramolecular or intermolecular bonding; thus, CMC might dissolve in water quickly, but it is difficult for the hydrophobic PDLLA particles to be resuspended. The manufacturer of AestheFill recommends vortexing the PDLLA particles for reconstitution, but the energy needed may not be sufficient. Although Chen et al. have established a back-and-forth method to accelerate reconstitution, this requires special tools, which is inconvenient [24]. In this study, emulsification was used to incorporate PDLLA and CMC. It is possible that the carboxymethyl groups of CMC interact with water to dissolve quickly, and intra/intermolecular hydrogen bonding between PDLLA and CMC could facilitate breaking the PDLLA particles into smaller microspheres. Therefore, the currently used PDLLA particles exhibit good solubility. The FTIR results demonstrated that there were no additional functional groups established (Figure 3). Thus, whether PDLLA and CMC can indeed encourage intra/intermolecular hydrogen bonding and whether this is the main reason for quick reconstitution require further investigation.

### 3.3. Implanting PDLLA Particles into Rabbit Dorsal Ears

**Figure 4:**
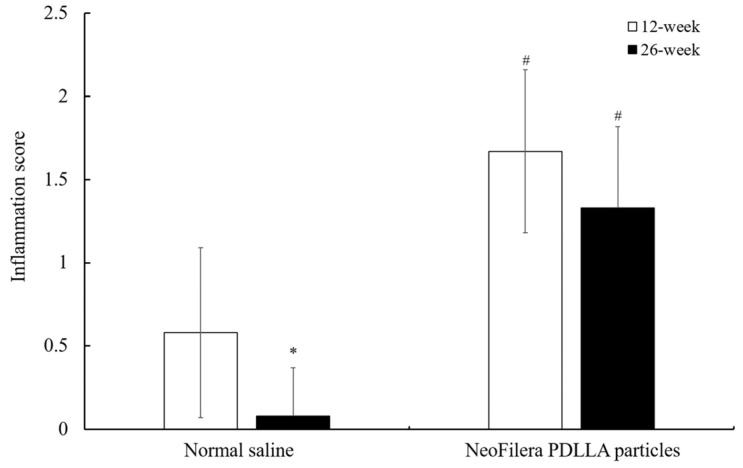
Inflammation scores of injected sites in normal saline or NeoFilera PDLLA particle groups at 12 weeks (white bars) or 26 weeks (black bars) post-injection. * *p* < 0.05 when comparing inflammation scores in normal saline at 12 weeks vs. 26 weeks post-injection. # *p* < 0.05 when comparing inflammation scores in normal saline vs. NeoFilera PDLLA particle groups at same time point.

When injecting normal saline or NeoFilera PDLLA particles into the rabbits’ ears, normal saline did not cause serious inflammation, as determined by the score (Figure 4). The inflammation score was 0.58 ± 0.51 and 0.08 ± 0.292 at 12 weeks and 26 weeks post-injection, respectively. The inflammation score at 26 weeks was much lower than that at 12 weeks post-injection. In contrast, the inflammation score was 1.67 ± 0.49, and it was much higher in NeoFilera PDLLA particle-injected animals at 12 weeks post-injection. Although the inflammation score was reduced to 1.33 ± 0.49 at 26 weeks when injecting NeoFilera PDLLA particles, it was still higher than the score in the normal saline group (Figure 4), suggesting that the PDLLA particles induced inflammation.

**Figure 5:**
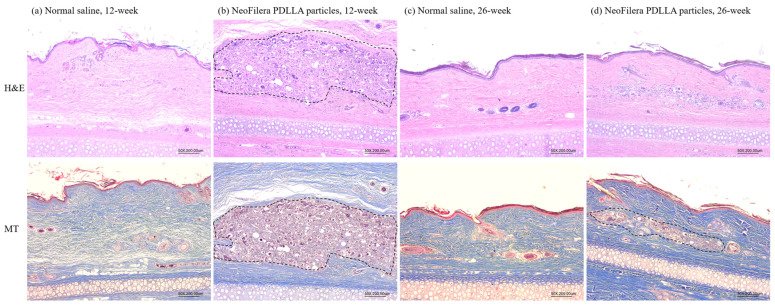
Histological analysis of hematoxylin and eosin (H&E) or Masson’s trichrome (MT) staining after injecting normal saline (**a**,**c**) or NeoFilera PDLLA particles (**b**,**d**) for 12 or 26 weeks.

The histological results showed that, at 12 weeks post-injection, fibroblast proliferation was observed when injecting NeoFilera PDLLA particles (area inside the black dashed line, Figure 5b). Fibroblast proliferation was not observed when injecting normal saline (Figure 5a). At 26 weeks post-injection, the ear tissue was more affected in the control animals (Figure 5c). The tissue also became more affected when injecting NeoFilera PDLLA particles. Proliferated fibroblasts were still observed (area inside the black dashed line, Figure 5d), as caused by the PDLLA particles.

When PLA particles are injected into the skin, the immune cells consider PLA as a foreign body and inflammation occurs, resulting in PLA-induced collagen synthesis [10,16]. Indeed, the inflammation score was higher when injecting the commercial NeoFilera PDLLA dermal fillers into the rabbits’ dorsal ears, while it was much lower in the normal saline group (Figure 4). Fibroblast proliferation was also observed in the NeoFilera PDLLA particle-injected area, suggesting that collagen formation occurred (Figure 5). H&E and MT staining can only stain the nucleus, cytoplasm, and intracellular matrix; thus, specific collagen formation should be confirmed using picrosirius red dye [39]. In addition, both NeoFilera and currently used PDLLA particles prepared by various stirring speeds displayed a smooth surface and a round shape. Particles with rough surfaces and irregular shapes have been shown to induce a foreign-body granuloma [40], suggesting that those with smooth surfaces and regular shapes are more suitable for dermal fillers. Although the soft-tissue augmentation function of the PDLLA particles generated in this study has not yet been investigated, all the physical–chemical properties shown here suggest their potential for use as a dermal filler.

## 4. Conclusions

Poly(D,L-lactic acid) (PDLLA) particles were prepared by emulsification in this study, and the relationship between the stirring speed and the physical–chemical properties of PDLLA particles was investigated. The results showed that a higher stirring speed resulted in smaller particles, but the stirring speed did not affect the molecular weight, optical rotation, pH value, and osmotic pressure of the PDLLA particles. The sizes of PDLLA particles generated using 100 and 200 rpm stirring speeds range between 20 and 100 μm, which may reduce the risk of phagocytosis. In addition, the reconstitution time of the PDLLA particles was less than 7 min, suggesting that they easily dissolve and disperse in the solution, resulting in the potential prevention of clinical complications. The in vivo experiment demonstrated that a rabbit dorsal ear model can be applied to analyze the soft-tissue augmentation function of PDLLA particles. The results presented in this study suggested that the PDLLA particles prepared at various stirring speeds exhibited similar physical–chemical properties to the currently used PDLLA dermal filler product and thus have potential for application as dermal fillers.

## Figures and Tables

**Table 1:** The physical–chemical properties of the PDLLA particles generated at various stirring speeds or in NeoFilera.

	Stirring Speed or Control	100 rpm	200 rpm	400 rpm	NeoFilera (Control)
Physical–Chemical Property					
Molecular weight of PDLLA (Daltons)		84,407 ± 812.9	84,386 ± 532.2	84,525 ± 339.0	84,352.7 ± 458.35
Optical rotation		+0.21 ± 0.03	+0.26 ± 0.02	+0.25 ± 0.05	+0.23 ± 0.04
pH value		7.00 ± 0.00	7.02 ± 0.00	7.02 ± 0.01	7.02 ± 0.00
Osmotic pressure (mOsm/kg)		280 ± 19.4	280 ± 18.8	280 ± 19.1	269 ± 2.0
Reconstitution (adding 1.4 mL distilled water) (′ represents minutes and ″ represents seconds)		5′26″	6′10″	5′58″	5′40″
Reconstitution (adding 8.0 mL distilled water) (′ represents minutes and ″ represents seconds)		2′13″	2′10″	2′34″	2′03″

## Data Availability

Data are contained within the article or Supplementary Material.

## Supplementary Materials

The following supporting information can be downloaded at: https://www.mdpi.com/article/10.3390/polym16233395/s1, Figure S1: The GPC curves of all PDLLA particles.
