# Supplementary material for: Investigating the Relationship Between the Emulsification Parameters and Physical–Chemical Properties of Poly(D,L-lactic acid) Particles for Dermal Fillers
Source: Polymers (Basel). 2024 Dec 1;16(23):3395. doi: 10.3390/polym16233395 (PMC11644387; doi:10.3390/polym16233395)
Supplement: Supplementary file 1 [file polymers-16-03395-s001.zip › polymers-3324767-supplementary.pdf]

Supplementary data

Investigating the relationship between the emulsification parameters and the physical–chemical properties of poly(D,L-lactic acid) particles for dermal fillers

Chen-Ying Su [1,2], You-Cheng Chang [1,2], Bo-Rong Lu [1,2], and Hsu-Wei Fang [1,2,3,*]

1 Department of Chemical Engineering and Biotechnology, National Taipei University of Technology. 1, Sec. 3, Zhongxiao E. Rd., Taipei 10608, Taiwan; chenying.su@mail.ntut.edu.tw (C.-Y. S.); youchengchang@mail.ntut.edu.tw (Y.-C. C.); t112738002@ntut.org.tw (B.-R. L.)

2 High-value Biomaterials Research and Commercialization Center, National Taipei University of Technology. 1, Sec. 3, Zhongxiao E. Rd., Taipei 10608, Taiwan.

3 Institute of Biomedical Engineering and Nanomedicine, National Health Research Institutes. No. 35, Keyan Road, Zhunan Town, Miaoli 35053, Taiwan.

*Correspondence: hwfang@ntut.edu.tw

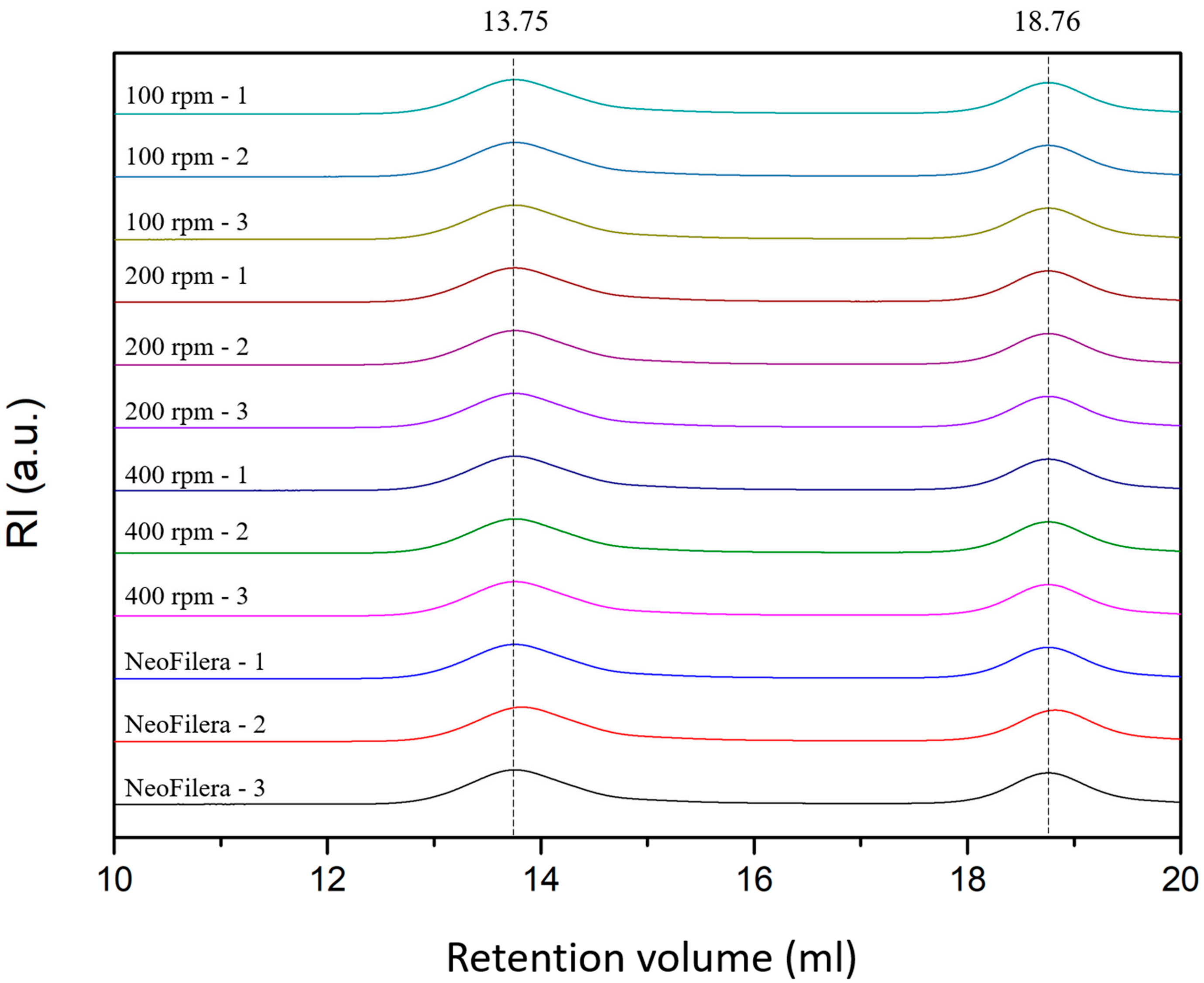

Figure S1. The gel permeation chromatography (GPC) curves of all PDLLA particles. The samples include individual PDLLA particle sample that is generated with 100, 200, or 400 rpm of stirring speed, and three commercial NeoFilera PDLLA dermal fillers. 13.75 ml represents the retention volume of PLA while 18.76 ml represents the retention volume of CMC.
